# The efficacy of detecting arrhythmia is higher with 7‐day continuous electrocardiographic patch monitoring than with 24‐h Holter monitoring

**DOI:** 10.1002/joa3.12865

**Published:** 2023-05-08

**Authors:** Ju Young Kim, Il‐Young Oh, Hyejin Lee, Ji Hyun Lee, Youngjin Cho, Yeongjoon Gil, Sunghoon Jung, Dae In Kim, Myung Geun Shin, Joo Yeon Yoo, Jeong Yeon Kwak

**Affiliations:** ^1^ Department of Family Medicine Seoul National University Bundang Hospital Seongnam Korea; ^2^ Department of Internal Medicine Seoul National University Bundang Hospital Seongnam Korea; ^3^ HUINNO Co., Ltd. Seoul Korea

**Keywords:** ambulatory electrocardiography monitoring, cardiac arrhythmias, Holter monitoring, wearable electronic devices

## Abstract

**Background:**

Detecting high‐risk arrhythmia is important in diagnosing patients with palpitations. We compared the diagnostic accuracies of 7‐day patch‐type electrocardiographic (ECG) monitoring and 24‐h Holter monitoring for detecting significant arrhythmias in patients with palpitations.

**Methods:**

This was a single‐center prospective trial with 58 participants who presented with palpitations, chest pain or syncope. Outcomes were defined as the detection of any one of six arrhythmias, including supraventricular tachycardia (SVT), atrial fibrillation or atrial flutter lasting more than 30 s, pauses of more than 3 s, high‐degree atrioventricular block, ventricular tachycardia (VT) >3 beats, or polymorphic VT/ventricular fibrillation. The McNemar test for paired proportions was used to compare arrhythmia detection rates.

**Results:**

The overall arrhythmia detection rate was higher with 7‐day ECG patch monitoring than with 24‐h Holter monitoring (34.5% vs. 19.0%, *p* = .008). Compared with the use of 24‐h Holter monitors, the use of 7‐day ECG patch monitors was associated with higher detection of SVT (29.3% vs. 13.8%, *p* = .042). No serious adverse skin reactions were reported among the ECG patch‐monitored participants.

**Conclusions:**

The results suggest that a 7‐day patch‐type continuous ECG monitor is more effective for the detection of supraventricular tachycardia than is a 24‐h Holter monitor. However, the clinical significance of device detected arrhythmia should be consolidated.

## Graphical abstract

A 7‐day patch‐type continuous electrocardiographic monitor is more effective in detecting significant arrhythmias than is a 24‐h Holter monitor.

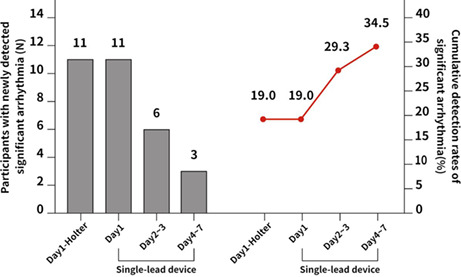

## 1 INTRODUCTION

Palpitations are one of the most common symptoms in patients who present to primary care clinicians and cardiologists.^1^ Although the causes are usually benign, palpitations occasionally manifest as potentially life‐threatening arrhythmias. Thus, an appropriate evaluation of palpitations is required.

Cardiac arrhythmias, including the development of new arrhythmias or significant changes in the rate of previously stable arrhythmias, are common causes of palpitations. Conventional Holter monitoring plays a significant role in the diagnosis of arrhythmias when evaluating palpitations.^2^ However, it has the drawbacks of low diagnostic yield in detecting paroxysmal arrhythmias, burdensome wires that interfere with daily activities, and the inability of some patients to activate the event recorders when symptoms occur.^3^, ^4^

Recently, several newer generation electrocardiography (ECG) monitoring devices with advanced technologies have shown more advantages over the conventional 24‐h Holter monitoring devices in terms of convenience, efficient energy use, longer duration of monitoring, wireless data transfer, and no interruption of daily activities.^5^ Researchers developed a deep neural network to diagnose cardiac arrhythmia, demonstrating its superior ability to classify the 12 rhythm classes compared with interpretations by individual cardiologists.^6^ Detection rates of cardiac arrhythmias for extended durations with fully automated and highly accurate systems were studied, and the results suggested that they could aid cardiologists in the accurate detection of arrhythmias.^7^, ^8^

**FIGURE 1:**
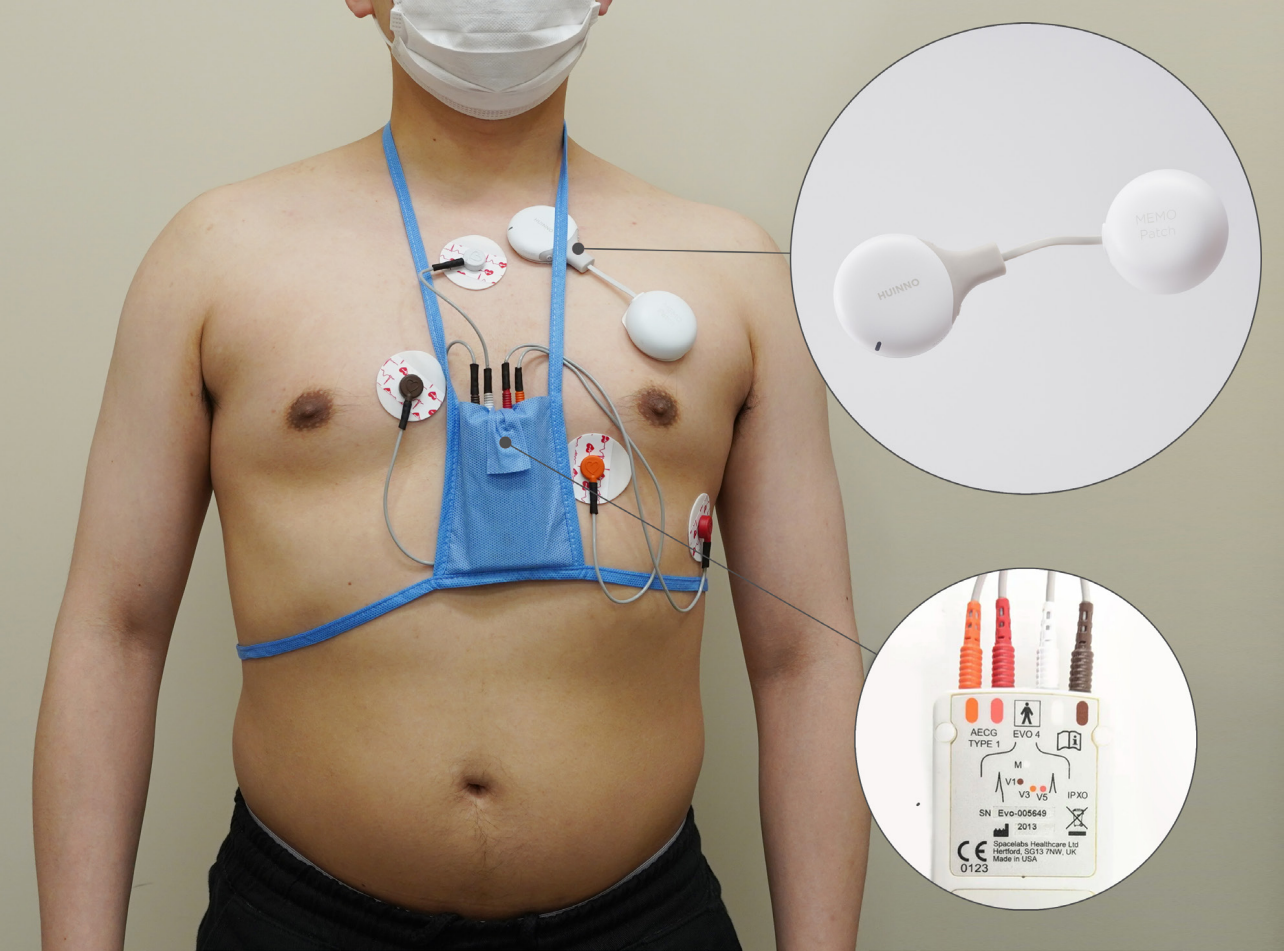
Overview of where the concomitant Holter electrocardiography and MEMO® patch is worn on the torso.

The MEMO patch version 1 (HUINNO Co., Ltd.) is the first Korean Food and Drug Administration (KFDA)‐approved, single‐lead, lightweight, 7‐day ambulatory ECG adhesive patch monitor (Figure 1). Soon after the release of version 1, version 2 was developed, tested, and approved for 14‐days of ambulatory ECG monitoring by KFDA. The device has no wires and can be re‐attached every day with disposable 3M adhesives; therefore, it does not interfere with the patient's daily activities.

We compared the diagnostic accuracies of 7‐day adhesive patch monitoring (the MEMO patch version 1) and 24‐h Holter monitoring for detecting arrhythmias in patients with palpitations.

## 2 METHODS

### 2.1 Study design and population

The Institutional Review Board of Seoul National University Bundang Hospital approved the protocol (approval No: E‐1901‐516‐001), and all participants provided informed consent to participate before enrollment. This was a single‐center, prospective cohort study. Between March 2021 and August 2021, 70 patients who visited the family medicine clinic for symptoms of palpitations, chest pain, or syncope or were referred to the cardiology department for ambulatory ECG monitoring were screened for eligibility. Patients were enrolled if they were >19 years old, capable of providing voluntary informed consent, and able to adhere to the study protocol during the 7 days of keeping a MEMO patch for ECG monitoring. Patients were excluded if they had undergone or were scheduled to undergo direct current cardioversion or catheter or surgical ablation procedures during the monitoring period due to underlying arrhythmias, or if they had known allergic reactions to adhesive patches. Of the 70 participants screened, 60 were enrolled in this study.

### 2.2 Study protocol and outcomes

A three channel Holter monitor (EVO; SPACELABS Healthcare Co.) was used for the first 24 h and the MEMO patch was concomitantly fitted over the left pectoral area of each participant's chest by clinical staffs as shown in Figure 1. The participants were given a symptom diary to record the suspected symptoms of arrhythmias and the time of symptom onset and finish. The participants were also instructed to wear the MEMO patch monitor for up to 7 days. On day 7, the participants returned the MEMO patch monitor via a prepaid mail package to the laboratory. After 1 week of monitoring, the participants visited the outpatient clinic for the results of the Holter and MEMO patch monitors and completed a survey regarding overall ease of use and satisfaction.

The MEMO patch can store ECG data for up to 14 days in its internal storage. The stored ECG data can be extracted by connecting the device to a dedicated cradle with software for data download. The extracted ECG data are automatically uploaded to a cloud‐based system through the software, and the initial analysis by the artificial neural network, constructed and trained by HUINNO, is automatically triggered. The network used in this study for the seven‐class ECG classification was based on the 152‐layer convolutional neural network architecture with skip connections, which is called a residual network, and squeeze and excitation blocks.^9^ The ECG recording was initially analyzed using deep learning‐based automated algorithms,^9^ however, technicians manually double checked and reviewed the artificial intelligence (AI) diagnosis and if needed, revised the diagnosis after it was confirmed by cardiologists. The correction rate of the AI driven diagnosis, except for the ECG noise, included a total of three diagnoses (3/51, 5.9%), specifically, two for second‐degree (Mobitz type I) atrioventricular block (AVB) and one for intermittent 2:1 AVB.

The network was trained by cardiologist‐reviewed 18 000 lead II ECG data recordings acquired from the Seoul National University Bundang Hospital.

The Holter and MEMO data were subjected to a technical review for report generation and quality assurance. This report was then uploaded to a secure website for independent review by two investigators (J.H.L. and Y.C.). If there were any discrepancies in the interpretation of the ECG signals, then the sets of signals were sent to a senior cardiologist who decided on the final classification (I.‐Y.O.).

Outcomes were defined as the detection of any one of the six arrhythmias: (1) supraventricular tachycardia (>3 beats, not including atrial fibrillation or flutter), (2) atrial fibrillation or atrial flutter lasting more than 30 s, (3) pause of more than 3 s, (4) AVB (second‐degree AVB Mobitz type II or third‐degree AVB), (5) ventricular tachycardia >3 beats, or (6) polymorphic ventricular tachycardia or ventricular fibrillation.

### 2.3 Statistics

The MEMO Patch and the Holter device were used simultaneously on the same patients during the first 24 h. This study compared the detection rates of arrhythmia events between the MEMO patch and Holter device over the total monitoring time of each device. Continuous variables are presented as means and standard deviations, and categorical variables are shown as numbers with percentages. The McNemar test for paired proportions was used to compare the detection rate for arrhythmias between the Holter and MEMO patch monitors.

#### 2.3.1 Estimation of sample size calculation

A previous study showed that the adhesive patch monitor detected 36 more arrhythmia events than did the Holter monitor, while the Holter monitor detected one event undetected by the adhesive patch monitor.^10^ Another study found that 202 atrial fibrillation or flutter episodes were detected in six patients with 14‐day ECG patches, while only one atrial fibrillation episode was detected in a patient with a 24‐h Holter monitor.^11^ Since our study aimed to compare 7‐day ECG patch monitors with 24‐h Holter monitors, we assumed that the odds ratio of detecting arrhythmias could be six times higher with the MEMO patch than with the 24‐h Holter monitor. A sample size of at least 57 after attrition achieved 90% power for a two‐tailed McNemar test with a type 1 error of 0.05 (G*Power software version 3.1.9.4).^12^

## 3 RESULTS

Of the 70 participants screened, 60 were enrolled; 2 enrolled patients were lost to follow‐up. A total of 58 participants with data available from both the Holter and MEMO patch groups were included in the final analysis. The baseline characteristics of the patients are presented in Table 1. The mean age was 50.5 years and 57% (33/58) were women. Hypertension (15/58, 26%) and dyslipidemia (14/58, 24%) were common underlying medical conditions.

**TABLE 1:** Baseline characteristics of participants (*N* = 58).

Characteristics	
Age (year), mean (SD)	50.03 (15.07)
Female, *n* (%)	33 (56.9)
Body mass index, kg/m^2^, mean (SD)	23.85 (3.72)
Comorbidities, *n* (%)	
Hypertension	15 (25.9)
Dyslipidemia	14 (24.1)
Diabetes mellitus	4 (6.9)
Aortic dissection	1 (1.7)
Transient ischemic attack	1 (1.7)
Concurrent medication, *n* (%)	
Antihypertensive medication	26 (44.8)
Lipid‐lowering medication	17 (29.3)
Psycholeptic treatment	5 (8.6)
Anti‐diabetic medication	4 (6.9)
Sex hormone treatments	4 (6.9)
Antithrombotic agents	3 (5.2)
Anti‐inflammatory agents	3 (5.2)

Abbreviation: SD, standard deviation.

### 3.1 Detection rate for significant arrhythmias

**FIGURE 2:**
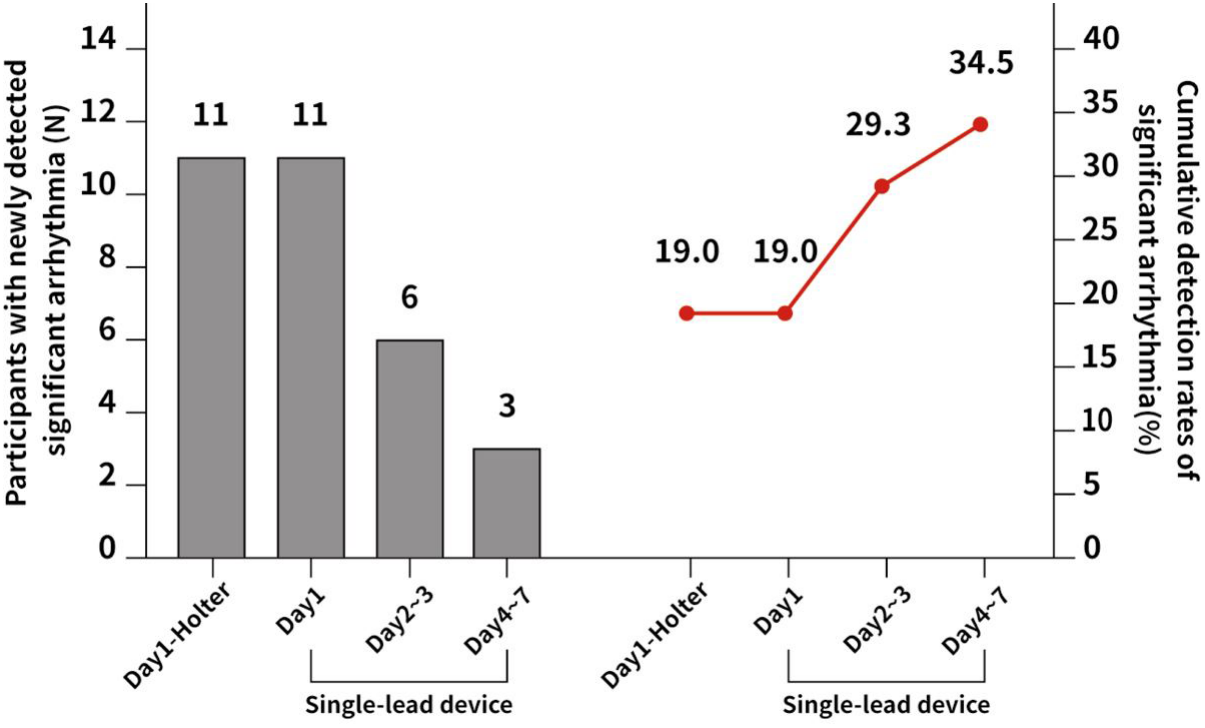
Detection and cumulative rates of significant arrhythmias using 24‐h Holter and 7‐day MEMO patch monitors.

The detection rates of significant arrhythmias, defined as the outcomes, are summarized in Table 2. The cumulative detection rates of MEMO patches on days 1 and 7 were 19.0% (11/58) and 34.5% (20/58), respectively, and that of Holter monitors on day 1 was 19.0%. Of the 44 participants without arrhythmias in 24‐h Holter monitoring, nine had significant arrhythmias in 7‐day MEMO patch monitoring (Figure 2).

**TABLE 2:** Detection of significant arrhythmias with a 24‐h Holter monitor versus a 7‐day MEMO patch monitor.

	MEMO patch monitor	24‐h Holter monitor	*p*‐value
Number of participants diagnosed with significant arrhythmias	20/58 (34.5%)	11/58 (19.0%)	.008
Number of detected arrhythmias	25	11	.005
Supraventricular tachycardia	17	8	.042
Atrial fibrillation/atrial flutter	6	2	.143
Sinus pause (≥3 s)	1	1	1.000
Second (Mobitz type II)/third‐degree AVB	0	0	‐
Ventricular tachycardia (≥3 events)	1	0	.315
Polymorphic ventricular tachycardia/ventricular fibrillation	0	0	‐

Abbreviation: AVB, atrioventricular block.

Among the 58 participants, six atrial fibrillation/atrial flutter episodes were detected by MEMO patch monitoring, and only two were detected by Holter monitoring.

The detection rate for total significant arrhythmias was higher with MEMO patches (34.5% vs. 19.0%, *p* = .008), as shown in Table 2.

### 3.2 Detection of all arrhythmias

A total of 51 arrhythmias were detected by MEMO patch monitoring and 22 arrhythmias were detected by Holter monitoring, as shown in Table 3. Supraventricular tachycardia was the most commonly detected arrhythmia (29.3% by the MEMO patch and 13.8% by the Holter monitor, *p* = .042).

**TABLE 3:** Detection of total arrhythmia events with a 24‐h Holter monitor versus a 7‐day MEMO patch monitor.

	MEMO patch monitor	24‐h Holter monitor	*p*‐value
Number of participants diagnosed with arrhythmias	29/58 (50.0%)	17/58 (29.3%)	.001
Number of detected arrhythmias	51	22	<.001
Supraventricular tachycardia	17	8	.042
Atrial fibrillation/atrial flutter	6	2	.143
Atrial premature beats	4	4	1.000
Ventricular premature beats	5	5	1.000
Tachycardia‐bradycardia syndrome	1	0	.315
Sinus pause (≥3 s)	1	1	1.000
Second‐degree (Mobitz type I) AVB	4	2	.402
Second‐degree (Mobitz type II) AVB	0	0	‐
Intermittent 2:1 AVB	1	0	.315
Third‐degree AVB	0	0	‐
Wolff–Parkinson–White syndrome	1	0	.315
Sick sinus syndrome	1	0	.315
Short run of accelerated idioventricular rhythm	1	0	.315
Ventricular tachycardia (≥3 events)	1	0	.315
Polymorphic ventricular tachycardia/ventricular fibrillation	0	0	‐

Abbreviation: AVB, atrioventricular block.

**FIGURE 3:**
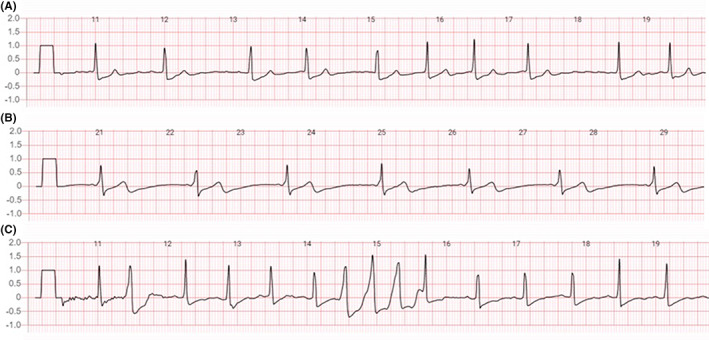
Examples of significant arrhythmias detected by a 7‐day electrocardiography monitor. (A) Electrocardiogram of a patient with asymptomatic atrial fibrillation. (B) Electrocardiogram of a patient with Wolff–Parkinson–White syndrome; (C) Electrocardiogram of a patient with ventricular tachycardia.

Notably, tachycardia‐bradycardia syndrome, Wolff–Parkinson–White syndrome, sick sinus syndrome, short run of accelerated idioventricular rhythm, and ventricular tachycardia were only detected using the MEMO patch. Several examples of arrhythmias detected by the MEMO patch are presented in Figure 3.

### 3.3 Adverse skin reactions

There were 99 reported adverse skin reactions among 9% (5/58) of the participants. Approximately 86% (85/99) of symptoms were self‐limited itching sensations with mild redness, which were categorized as mild reactions; 82% (81/99) of all cases occurred 24 h after adhesive patches were attached to the participants' skins. No severe reactions were observed in this study.

## 4 DISCUSSION

Our study demonstrated that 7 days of monitoring with a wearable ECG patch was superior to 24 h of monitoring with a Holter monitor for detecting clinically significant supraventricular tachycardia among patients with palpitations, chest pain, or syncope. However, 7 days of monitoring failed to detect other clinically significant arrhythmias including atrial fibrillation more effectively than a Holter monitor in our study participants.

Both MEMO patch monitoring and Holter monitoring showed similar arrhythmia detection rates over 24 h. Compared to 24‐h Holter monitoring, the detection rate increased 1.24‐fold during the extended 6 days of MEMO patch monitoring.

The detection rate with 14‐day Zio patch monitoring (iRhythm Technologies, Inc.) was higher than that with 24‐h Holter monitoring (96 vs. 61 events, *p* < .001).^10^ Another study conducted by Chua et al. showed that 14 days of patch‐type ECG monitoring detected more arrhythmia events than did 24‐h Holter monitoring (66% vs. 9%, *p* < .001).^11^ Another study also confirmed that 14‐day ECG patch monitoring showed higher arrhythmia detection rates (up to 60% [94/158]) than did 24‐h Holter monitoring (up to 19%).^13^ The diagnostic yield of arrhythmias usually increases proportionally with the monitoring duration. However, several studies showed that approximately 90% of total arrhythmias can be detected if ECG monitoring periods are between 7 and 10 days.^11^, ^13^ In our study, 7‐days of ECG patch monitoring among generally low risk patients with symptoms could yield a low detection rate of significant arrhythmias other than supraventricular tachycardia. Thus, a longer monitoring duration of 14 days may be needed to detect significant arrhythmias including subclinical atrial fibrillation/flutter.

Holter monitors are the device of choice for cardiac monitoring to detect arrhythmias. However, because of their size, inconvenience, and relatively short monitoring period, their usefulness in long‐term ambulatory monitoring is limited. Recently, many wearable devices have been introduced for monitoring patients remotely, which are small, comfortable, and compact. Several modalities of ECG monitoring exist, including monitoring type (continuous versus event monitoring), presence of event trigger function, presence of real‐time monitoring, or duration of monitoring.^14^ Compared with Holter monitors, patch‐type monitors consist of a single lead, are easier to use, and have higher adherence; however, they might suffer from low signal quality with different body types.^15^ Patch‐type ECGs usually do not have functions such as event‐based recording or real‐time data monitoring. In patients with intermittent palpations, patch‐type ECG monitoring may be appropriate for symptom rhythm correlations.

Among patients treated for atrial fibrillation, 72‐h single‐lead ECG monitoring was superior to 24‐h Holter monitoring; an additional 13.6% of patients with negative results in Holter monitoring were diagnosed with paroxysmal atrial fibrillation using 72‐h single‐lead ECG monitoring.^16^ Undiagnosed paroxysmal atrial fibrillation can lead to recurrent stroke or embolic events.^17^, ^18^ In addition, a higher atrial burden is a significant risk factor for clinical atrial fibrillation and future stroke.^19^ In this regard, continuous monitoring using patch‐type ECG can be useful, especially for patients with a history of strokes or transient ischemic attacks.^20^ Ambulatory patch‐type ECG monitoring may also be useful in emergency department patients with unexplained syncope.^21^

Smartphone‐based arrhythmia detection using a combined approach with single‐lead ECG and photoplethysmography is a promising tool for the early detection of atrial fibrillation.^22^ Due to their general availability, smartphone‐based algorithms enable patients to easily record symptomatic arrhythmias; however, short subclinical episodes may not be detected. A recent meta‐analysis showed that smartphones using only photoplethysmography‐based algorithms seemed to be biased, low‐quality monitoring devices with unrealistically high sensitivity and specificity.^23^

Other types of portable devices include the external loop recorder, which has a recording time of up to 4 weeks, and requires activation by patients during the onset of symptoms. In contrast, patch‐type ECG monitors have no external leads or wires, usually have a recording time of up to 14 days, and continuously monitor the patients' signals; thus, they are more useful in conditions such as syncope.^24^ Implantable loop recorders have the longest recording time of up to 3 years, activated by both an automatic algorithm and patient input; however, invasive procedures, high costs, and limited availability constrain their widespread use among patients.^24^ Various devices with different diagnostic yields, cost‐effectiveness, and patient preference and convenience should be considered when choosing diagnostic methods for symptomatic patients or screening high‐risk patients.

In our study, we also found that meaningful arrhythmias, including sick sinus syndrome and Wolff–Parkinson–White syndrome, were only detected in MEMO patch monitoring. Tachycardia bradycardia syndrome and sick sinus syndrome can be categorized as sinus node dysfunction, which can cause many symptoms, including syncope, presyncope, and lightheadedness, and patients with these conditions may need permanent pacemakers and concomitant treatment for atrial fibrillation.^25^ Wolff–Parkinson–White syndrome causes palpitations or syncope, and if accompanied by atrial fibrillation, it may cause ventricular fibrillation and sudden death, although that is rare.^26^

This study has several limitations. The participants were recruited from one hospital, which limits the generalizability of the study findings. Moreover, there might have been false‐positive or false‐negative episodes with the MEMO patch monitoring that could not be accounted for because of the lack of validated comparable tools. However, considering the same detection rates on the first day of concomitant Holter and MEMO patch monitoring, it can be inferred that the detection rates of both devices were similar. Further studies are required to confirm the effectiveness of 7‐day continuous ECG patch monitoring in detecting meaningful arrhythmias in specific populations.

## 5 CONCLUSION

In this single‐center prospective trial, 7‐day patch‐type continuous ECG monitoring was more effective in detecting supraventricular tachycardia than was 24‐h Holter monitoring. However, detecting other types of significant arrhythmias with ECG patch monitoring might require a longer duration of more than 7 days. Further studies on the efficacy of detecting specific arrhythmias in certain populations are required. In addition, studying the comparative effectiveness will help choose the most appropriate options for patients in terms of monitoring periods, costs, convenience, and limitations among various types of ECG devices.

## AUTHOR CONTRIBUTIONS

Ju Young Kim conceived, designed, and performed the study. Ji Hyun Lee, Youngjin Cho, and Hyejin Lee designed and performed the study and reviewed the literature. Yeongjoon Gil and Sunghoon Jung performed the artificial intelligence analytic program and were involved in the interpretation of the results. Dae In Kim, Joo Yeon Yoo, and Jeong Yeon Kwak performed the clinical study and collected clinical data. Dae In Kim, Myung Geun Shin, and Jeong Yeon Kwak performed statistical analyses. Il‐young Oh took part in the study design and was responsible for the final draft. All authors have contributed to the manuscript and approved the submitted version.

## FUNDING INFORMATION

This work was mainly supported by the Korea International Cooperation Agency under the title “Prediction and management system for the patients of cardiovascular disease” between 2018 and 2021 (No. 2018‐1580). This study was also partly supported by the Korea Medical Device Development Fund from the Ministry of Science and ICT (Project number: 1711138361), Ministry of Trade Industry and Energy (Project number: RS‐2020‐KD00017), Ministry of Health & Welfare (Project number: 1711139106), and Ministry of Food and Drug Safety (Project Number: RS‐2021‐KD000011). The funders had no role in study design, data collection and analysis, decision to publish, or preparation of the manuscript.

## CONFLICT OF INTEREST STATEMENT

JuYK is employed part‐time at HUINNO Co., Ltd Seoul, Korea. YG, SJ, DK, MGS, JYY, and JK are currently employed at HUINNO Co., Ltd Seoul, Korea. The rest of the authors declare that they have no conflicts of interest.

## DECLARATIONS

*Approval of the research protocol*: The Institutional Review Board of Seoul National University Bundang Hospital approved the protocol on February 08, 2019. *Informed consent*: All participants provided informed consent to participate before enrollment. *Registry and the Registration No*: Approval No: E‐1901‐516‐001. *Animal studies*: N/A.

## ETHICS APPROVAL STATEMENT

The Institutional Review Board of Seoul National University Bundang Hospital approved the protocol (approval No: E‐1901‐516‐001).

## PATIENT CONSENT STATEMENT

All participants provided informed consent to participate before enrollment.

## Data Availability

The data that support the findings of this study are available from the corresponding author (Il‐Young Oh) upon reasonable request.
